# Boosting corrects a memory B cell defect in SARS-CoV-2 mRNA–vaccinated patients with inflammatory bowel disease

**DOI:** 10.1172/jci.insight.159618

**Published:** 2022-06-22

**Authors:** Kathryn A. Pape, Thamotharampillai Dileepan, William E. Matchett, Charles Ellwood, Samuel Stresemann, Amanda J. Kabage, Daria Kozysa, Clayton Evert, Michael Matson, Sharon Lopez, Peter D. Krueger, Carolyn T. Graiziger, Byron P. Vaughn, Eugenia Shmidt, Joshua Rhein, Timothy W. Schacker, Tyler D. Bold, Ryan A. Langlois, Alexander Khoruts, Marc K. Jenkins

**Affiliations:** 1 Department of Microbiology and Immunology,; 2 Center for Immunology,; 3 Department of Medicine, Division of Gastroenterology, and; 4 Department of Medicine, Division of Infectious Disease, University of Minnesota Medical School, Minneapolis, Minnesota, USA.

**Keywords:** COVID-19, Immunology, Adaptive immunity, Inflammatory bowel disease, Memory

## Abstract

Immunosuppressed patients with inflammatory bowel disease (IBD) generate lower amounts of SARS-CoV-2 spike antibodies after mRNA vaccination than healthy controls. We assessed SARS-CoV-2 spike S1 receptor binding domain–specific (S1-RBD–specific) B lymphocytes to identify the underlying cellular defects. Patients with IBD produced fewer anti–S1-RBD antibody–secreting B cells than controls after the first mRNA vaccination and lower amounts of total and neutralizing antibodies after the second. S1-RBD–specific memory B cells were generated to the same degree in IBD and control groups and were numerically stable for 5 months. However, the memory B cells in patients with IBD had a lower S1-RBD–binding capacity than those in controls, which is indicative of a defect in antibody affinity maturation. Administration of a third shot to patients with IBD elevated serum antibodies and generated memory B cells with a normal antigen-binding capacity. These results show that patients with IBD have defects in the formation of antibody-secreting B cells and affinity-matured memory B cells that are corrected by a third vaccination.

## Introduction

Inflammatory bowel disease (IBD), which includes ulcerative colitis and Crohn’s disease, affects as much as 1% of the population in the developed Western countries, and its incidence is rising rapidly globally (1, 2). Although treatments reduce IBD activity, they also have the potential to inhibit beneficial immune responses to microbes and vaccines. The use of anti-TNF therapy for IBD has been associated with attenuated serologic responses to SARS-CoV-2 in the course of COVID-19 infection (3), while use of corticosteroids and immunomodulators in patients with IBD has been associated with increased severity of COVID-19 (4, 5). Biologic therapies in patients with IBD, especially those targeting TNF, have also been associated with lower serologic responses to mRNA vaccines against SARS-CoV-2 (5–7). The cellular bases for these deficits in humoral immunity are unknown.

We performed an analysis by focusing on SARS-CoV-2 spike S1 receptor binding domain–specific (S1-RBD–specific) B cells in the peripheral blood of vaccinated individuals. S1-RBD was chosen for study because it facilitates viral entry by interacting with the ACE2 receptor on human lung epithelial cells (8) and, therefore, is a target of neutralizing antibodies (9). People who have never been exposed to S1-RBD via infection or vaccination contain rare naive B cells that display S1-RBD antibodies on the cell surface (10). After SARS-CoV-2 infection or vaccination, these B cells likely encounter S1-RBD in lymph nodes and receive signals from helper T cells that cause the B cells to proliferate and differentiate into short-lived antibody-secreting plasmablasts (11) or germinal center cells (12) that mutate the antigen-combining sites of their antibodies (13). B cells that acquire affinity-enhancing antibody mutations outcompete other B cells in the germinal centers and survive to become either long-lived memory cells that display their antibodies on the cell surface (14–16) or plasma cells that constitutively secrete antibodies and maintain serum antibody levels (17–19). After subsequent exposure to antigen, memory B cells proliferate rapidly and generate plasmablasts, which boost the amount of antigen-specific antibody in the serum to aid in antigen clearance or, to a lesser extent, become germinal center cells to generate new memory B cells with additional antibody mutations (20–22).

Effective vaccines generate plasmablasts and plasma cells as short- and long-lived sources of virus-neutralizing antibodies and affinity-matured memory B cells capable of rapid production of antibody-secreting cells after viral infection (23). The immunosuppressive drugs used to treat IBD could interfere with production of any or all of these B cell types. Therapies targeting TNF have special potential for deleterious effects on B cell responses because TNF is critical for the survival of stromal cells that organize the germinal center B cell competition that is critical for affinity maturation (24). Here, we sought to determine how well antibody-secreting plasmablasts and affinity-matured memory B cells are generated in patients with IBD after SARS-CoV-2 mRNA vaccination.

## Results

### Patients with IBD have a defect in S1-RBD antibody production after the first vaccination.

Thirty healthy volunteers and 42 patients with IBD on stable immunosuppressive treatments, none of whom had a prior exposure to SARS-CoV-2, were recruited into the study. Most of the patients with IBD were being treated with biologic agents targeting TNF (infliximab, adalimumab, or golimumab) alone, although some were receiving a blocker of IL-12/23 (ustekinumab), α_4_β_7_ integrin (vedolizumab), or combination therapy with an immunomodulator (Table 1).

**Figure 1:**
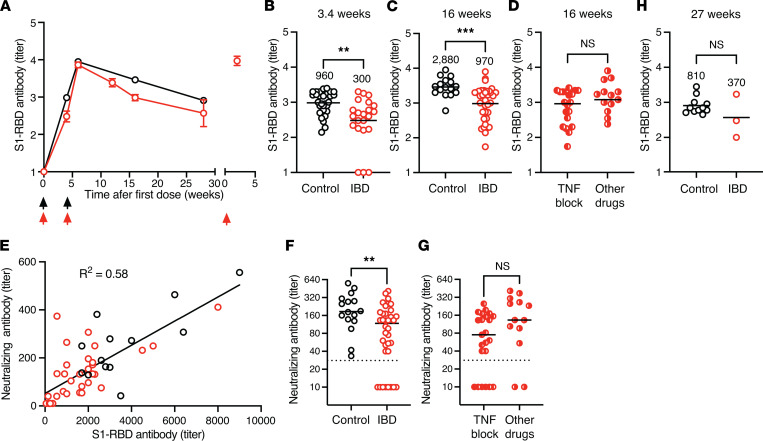
S1-RBD–specific antibody response after mRNA vaccination. (**A**) Mean log_10_ S1-RBD antibody titers (± SEM) for controls (black circles, *n* = 11–30) and patients with IBD (red circles, *n* = 3–36) at the indicated times (arrows) after the first, second, and third dose of mRNA vaccine. *x* axis values are the means of time intervals of 1–3 weeks. For example, a group with 5 samples at 15, 15, 16, 17, and 17 weeks would be assigned an *x* axis value of 16 weeks. (**B–D** and **H**) Log_10_ S1-RBD antibody titers from individual plasma samples from the indicated groups (**B**) 3.4, (**C**) 16, or (**H**) 27 weeks after the first vaccination. Mean titers are indicated with horizontal bars and a numerical value over each group. Values in each graph were compared with Student’s *t* test. (**E**) S1-RBD antibody titers versus neutralizing antibody titers for individual samples from controls (black circles, *n* = 16) and patients with IBD (red circles, *n* = 36) 16 weeks after the first vaccination, compared by linear regression. (**F** and **G**) Neutralizing antibody titers from individual samples from the indicated group 16 weeks after the first vaccination. Mean titers are indicated with horizontal bars. Values in each graph were compared with Student’s *t* test. (**B–D** and **F–H**) **P < 0.01, ***P < 0.001.

We first assessed S1-RBD–specific serum antibodies to confirm that the patients with IBD in our study had the serological defects noted in other publications (5, 6). As expected, based on the study entry criteria, no participants in either group had S1-RBD antibodies at the time of vaccination, indicating that they had not been exposed to SARS-CoV-2 in the past (Figure 1A). S1-RBD antibodies increased after the first dose of mRNA vaccine to an average titer of 960 in the control group and a significantly lower titer of 300 in the IBD group (Figure 1, A and B). The second vaccination at 3.4 weeks (arrived at by averaging Pfizer at 3 and Moderna at 4 weeks) resulted in a peak S1-RBD antibody titer of 8850 in the control group and 7280 in the IBD group at 6 weeks, which then fell to 2880 in the control group and 970 in the IBD group at 16 weeks (Figure 1, A and C). The S1-RBD antibody titers at 16 weeks of patients with IBD were significantly lower than those of the control group (Figure 1C). While patients treated with TNF blockers had slightly lower titers than those on other immunosuppressive drugs, this difference was not significant (Figure 1D). At 16 weeks, the amounts of total S1-RBD antibody and antibody capable of neutralizing SARS-CoV-2 in vitro were well correlated (Figure 1E). Patients with IBD had significantly fewer neutralizing antibodies than controls, and these were undetectable in 8 patients with IBD (Figure 1F), with a trend toward lower neutralizing titers in those treated with TNF blockers that was not statistically significant (Figure 1G). By 27 weeks, the S1-RBD antibody titer fell to 810 in the control group and to 370 in the IBD group. This difference was not statistically significant, likely because the IBD group contained only 3 patients, as the rest had received a third vaccine dose before a sample after the second vaccination could be collected (Figure 1, A and H). A third vaccination caused S1-RBD antibody levels in patients with IBD to increase 30-fold 6 weeks later (Figure 1A). Thus, the patients with IBD in this study had a defect in S1-RBD antibody production, as reported in other studies (5, 6). However, the S1-RBD antibody levels in patients with IBD were boosted to high levels after a third vaccination.

### Patients with IBD have a defect in the production of S1-RBD–specific plasmablasts.

**Figure 2:**
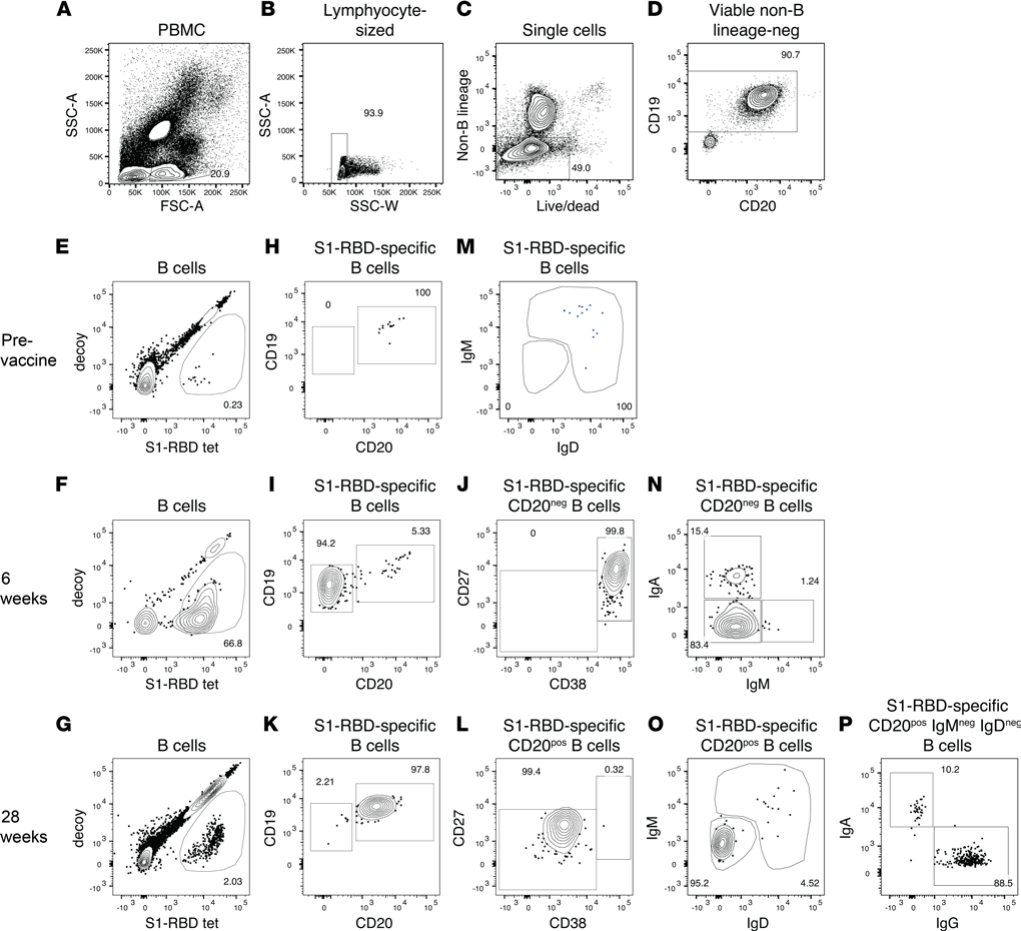
Identification of S1-RBD–specific B cells by flow cytometry. B cells were identified in PBMC samples by flow cytometry as cells with the (**A**) light scatter properties of lymphocytes and (**B**) a side scatter area versus width profile of singlets (**C**) that lacked B lineage–negative markers and did not bind a viability dye and (**D**) expressed CD19. (**E** and **F**) S1-RBD–binding B cells were identified as B cells that expressed low amounts of decoy and bound the S1-RBD tetramer. The frequencies of these cells are shown in the gates. (**E**) Examples of a sample before vaccination or (**F**) samples from a participant 5 weeks after the first vaccination and 1 week after the second or (**G**) 28 weeks after the first vaccination and 24.5 weeks after second are shown. S1-RBD–specific naive cells were identified as (**H**) CD19^+^CD20^+^ and (**M**) IgM^+^IgD^+^ cells. S1-RBD–specific plasmablasts were identified as (**I**) CD19^lo^CD20^‒^ and (**J**) CD27^hi^CD38^hi^ cells and (**N**) further divided by expression of IgA and lack of expression of IgM. S1-RBD–specific memory B cells were identified as (**K**) CD19^+^CD20^+^ and (**L**) CD27^+^CD38^lo^ cells and further divided by lack of expression of (**O**) IgM and IgD and (**P**) expression of IgA or IgG.

We assessed S1-RBD–specific B cells to determine the cellular bases for the serological defects observed in patients with IBD. Magnetic enrichment and flow cytometry were used to identify peripheral blood B cells with the capacity to bind a fluorophore-labeled S1-RBD tetramer (10). A tetramer was used to ensure detection of B cells with lower affinity surface antibodies than can be detected with antigen monomers (25), and cell enrichment was used to increase the sensitivity of detection (21, 26, 27). PBMCs were mixed with an S1-RBD tetramer labeled with the fluorochrome Alexa Fluor 647 (AF647), a decoy tetramer labeled with the fluorochrome phycoerythrin-conjugated AF647 (PE-AF647), and then with magnetic beads conjugated with AF647 antibodies. The mixture was then passed over a magnetized column and the bound, and unbound fractions were collected separately and stained with fluorochrome-labeled antibodies specific for markers of interest. The samples were then analyzed by flow cytometry and gated for viable single cells with the light scatter properties of lymphocytes that did not express CD3, CD14, or CD16 (Figure 2, A–C). B cells were identified in this population based on expression of CD19 (Figure 2D), and S1-RBD–specific B cells were identified as cells that bound the S1-RBD but not the decoy tetramer, as shown in Figure 2, E and F (for examples from participants before vaccination, see Figure 2E; 5 weeks after the first vaccination and 1 week after the second, see Figure 2F; or 28 weeks after the first vaccination and 24.5 weeks after the second, see Figure 2G). The S1-RBD–binding B cells were further divided into CD19^+^CD20^+^ naive B cells (Figure 2H), CD19^lo^CD20^–^ plasmablasts (28) (Figure 2I) that also expressed high levels of CD27 and CD38 (Figure 2J), or CD19^+^CD20^+^ memory B cells (Figure 2K) that were CD27^intermediate^CD38^lo^ (Figure 2L). Naive B cells were identified as IgM^+^IgD^+^ cells (Figure 2M). Plasmablasts were divided based on expression of IgA or as IgM^−^ cells, most of which likely expressed IgG (Figure 2N). IgG-expressing plasmablasts were identified in this way because of downregulation of surface IgG (29). Isotype-switched memory B cells were identified as cells that had lost IgM and IgD (Figure 2O) and expressed IgA or IgG (Figure 2P).

**Figure 3:**
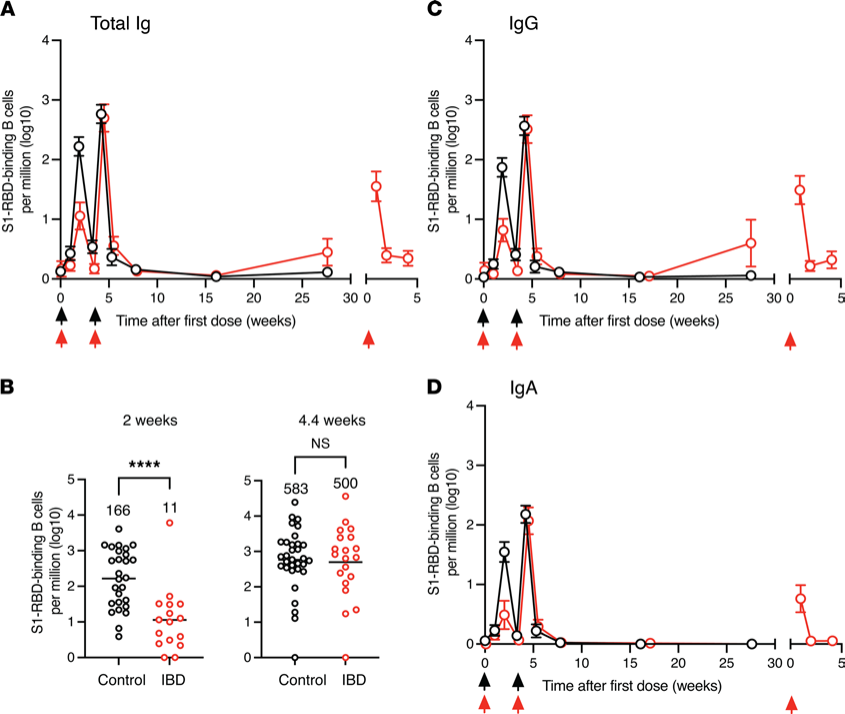
S1-RBD–specific plasmablast induction after mRNA vaccination. (**A**) Mean log_10_ numbers of S1-RBD tetramer–binding plasmablasts of all isotypes per million total B cells (± SEM) in the peripheral blood of controls (black circles, *n* = 12–30) or patients with IBD (red circles, *n* = 5–36) at the indicated times after vaccinations (arrows). Any cell per million value that was less than or equal to 1 was set to 0 on the log_10_ cell per million plots. Such values were at or below the limit of detection. Values on the *x* axis are the means of 1- to 3-week time intervals. (**B**) Log_10_ numbers of S1-RBD–specific plasmablasts from individual samples from the indicated groups. Groups were compared with Student’s *t* test. ****P < 0.0001. (**C** and **D**) Mean log_10_ number of S1-RBD tetramer–binding (**C**) IgA^+^ or (**D**) IgG^+^ plasmablasts per million total B cells (± SEM) in the peripheral blood of controls (black circles, *n* = 12–30) or patients with IBD (red circles, *n* = 5–36) at the indicated times after vaccinations (arrows).

This strategy was first used to determine the frequency S1-RBD–specific antibody-secreting plasmablasts. Before vaccination, controls and patients with IBD contained 12 ± 3 and 9 ± 4 S1-RBD tetramer–binding B cells per million total B cells (mean ± SD), respectively. These populations primarily consisted of unswitched naive B cells (Figure 2M) (on average, 69% and 60% IgM^+^IgD^+^ cells for controls and IBD groups, respectively), with fewer than 2 antibody-secreting plasmablasts per million total B cells (Figure 3A), as expected in individuals who had never been exposed to the SARS-CoV-2 spike protein. By 2 weeks after vaccination, the frequency of S1-RBD tetramer–binding plasmablasts increased to 166 per million total B cells in controls but only 11 per million total B cells in patients with IBD (Figure 3, A and B). The defect in patients with IBD applied to plasmablasts expressing IgG (Figure 3C) or IgA (Figure 3D). The reduction in plasmablasts matched the reduction in antibody levels observed in the IBD group a month after the first vaccination (Figure 1, A and B). The frequency of S1-RBD tetramer–binding plasmablasts in both groups fell dramatically at 3–4 weeks as expected for these short-lived cells (Figure 3A). S1-RBD tetramer–binding plasmablasts then spiked to 583 cells per million total B cells in the control group and 500 per million total B cells in the IBD group 1 week after the second vaccination (Figure 3, A and B) and then fell to 1 cell per million total B cells in both cases 2 weeks later (Figure 3A). Administration of a third dose of vaccine to the IBD group sometime after 12 weeks following the second dose caused plasmablasts to spike again to 180 cells per million total B cells and this burst contained cells that expressed IgG and IgA (Figure 3, C and D). Thus, patients with IBD have a defect in S1-RBD–specific plasmablast formation after the first vaccination that is less apparent after the second. In addition, a third vaccination stimulated patients with IBD to produce another round of plasmablasts.

### Patients with IBD generate the same frequency of S1-RBD–specific memory B cells as controls.

**Figure 4:**
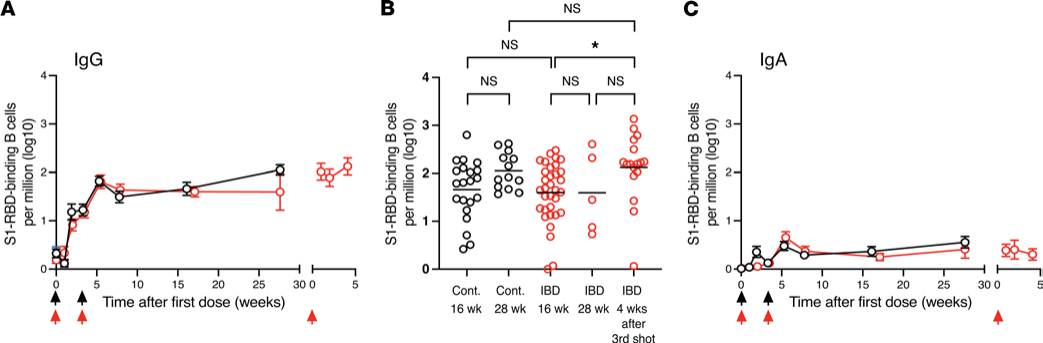
S1-RBD–specific memory B cell induction after mRNA vaccination. (**A**) Mean (± SEM) log_10_ numbers of S1-RBD tetramer–binding IgG memory B cells per million total B cells in the peripheral blood of controls (black circles, *n* = 12–30) or patients with IBD (red circles, *n* = 5–36) at the indicated times after vaccinations (arrows). (**B**) Log_10_ numbers of S1-RBD–specific IgG memory B cells from individual samples from the indicated groups. Groups were compared with ANOVA. *P < 0.05. (**C**) Mean (± SEM) log_10_ number of S1-RBD tetramer–binding IgA memory B cells per million total B cells in the peripheral blood of controls (black circles, *n* = 12–30) or patients with IBD (red circles, *n* = 5–36) at the indicated times after vaccinations (arrows). Any cell per million value that was less than or equal to 1 was set to 0 on the log_10_ cell per million plots. Such values were at or below the limit of detection. Values on the *x* axis are the means of 1- to 3-week time intervals.

We then enumerated S1-RBD–specific memory B cells, which form from naive B cells that survive the germinal center reaction in the secondary lymphoid organs and are likely the precursors of the plasmablasts observed in the blood after the second and third vaccinations (30). We focused on memory B cells that had undergone isotype switching. S1-RBD tetramer–binding IgG memory B cells increased in controls and patients with IBD from a baseline of 2 per million total B cells to a frequency of about 20 per million total B cells by 3–4 weeks after the first vaccination (Figure 4A). The second vaccination then caused the memory B cells in both groups to increase to about 80 cells per million total B cells at 6 weeks and fall slightly to 50 per million total B cells at 16 weeks. The memory B cell frequency in the control group increased to 100 per million total B cells between 16 and 28 weeks as noted in another study (16); however, this rise was not statistically significant (Figure 4B). Again, there were too few participants in the IBD group at 28 weeks to get an accurate memory B cell measurement. However, at 1 month after the third vaccination the IgG-expressing memory B cell frequency in the IBD group increased about 2-fold over that observed at 16 weeks. IgA memory B cells also increased after the first and second vaccinations in both the control and IBD groups but were much less abundant than IgG memory B cells (Figure 4C) and did not increase after the third vaccination.

Thus, S1-RBD–specific memory B cells formed at the same frequency in control and IBD participants at all times after the first and second vaccinations, and after a third vaccination patients with IBD had a similar frequency of memory B cells to that of controls.

### Patients with IBD have a defect in memory B cell affinity maturation that is corrected by a third vaccination.

**Figure 5:**
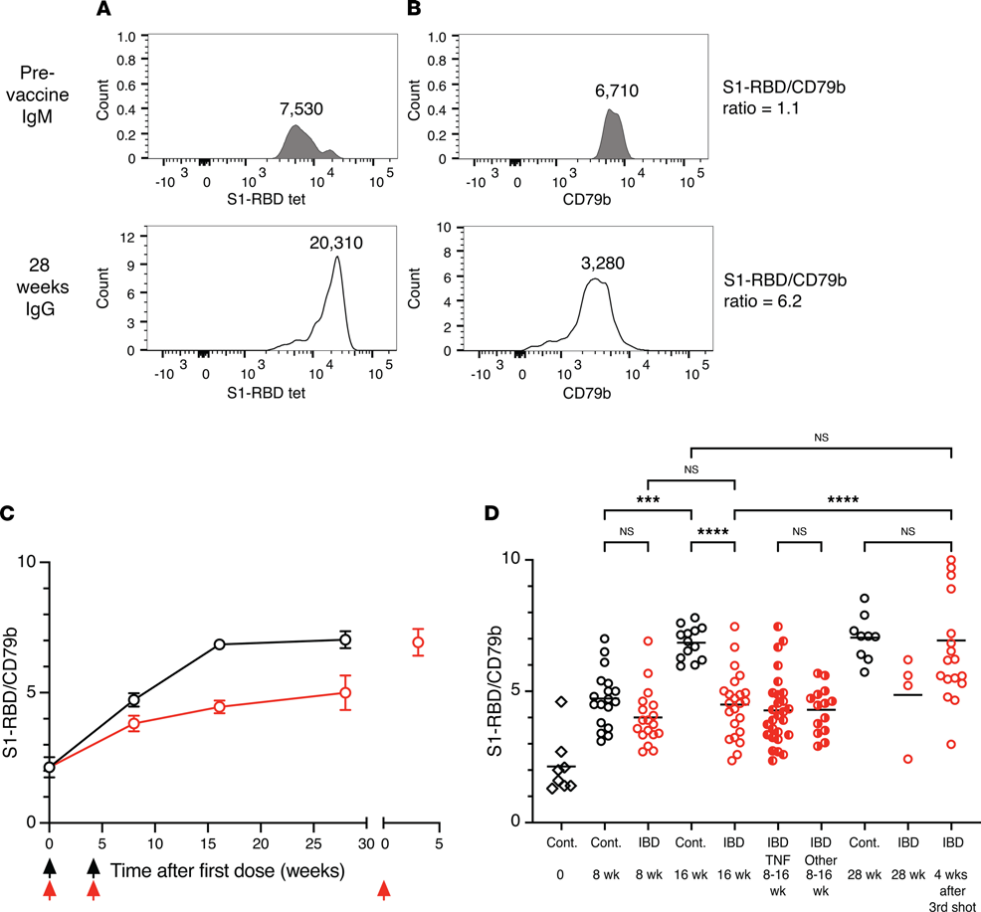
Affinity maturation by S1-RBD–specific memory B cells. (**A** and **B**) Flow cytometry plots of (**A**) S1-RBD tetramer or (**B**) CD79b staining of IgM^+^ naive B cells from a sample before vaccination (top) or IgG^+^ memory B cells from a sample 28 weeks after the first vaccination (bottom). Mean fluorescence intensity (MFI) values are shown above each histogram. (**C**) S1-RBD tetramer MFI/CD79b MFI ratios for IgM^+^ cells before vaccination or IgG^+^ cells from controls (black circles, *n* = 8–18) or patients with IBD (red circles, *n* = 5–26) after vaccinations (arrows). (**D**) S1-RBD tetramer MFI/CD79b MFI ratios from individual samples from the indicated groups. “TNF” refers to patients with IBD treated with TNF blockers and “Other” to patients with IBD treated with other immunosuppressants. To be used for this analysis a sample had to contain at least 10 S1-RBD–specific cells. For the time point before vaccination, there was 1 outlier that had an S1-RBD tetramer MFI/CD79b MFI ratio of 8 that was excluded from the analysis because subsequent 2-week and 4-week IgG time points from this individual had a ratio of less than 4. Values were compared with 1-way ANOVA. ***P < 0. 001, ****P < 0. 0001.

Although memory B cells formed at the same frequency in controls and patients with IBD, it remained possible that the quality of the cells in the 2 groups was different. Memory B cells are the progeny of germinal center B cells that fortuitously generate antibody mutations that increase the affinity for antigen and provide a survival advantage in a process called affinity maturation (17). It was of special interest to study affinity maturation in TNF inhibitor–treated patients with IBD because TNF is critical for germinal center formation (24). We assessed this possibility by determining how well the memory B cells in patients with IBD bound S1-RBD. Flow cytometry was used to measure the amounts of S1-RBD tetramer and CD79b, a component of the surface antibody complex (31), bound per B cell. Our previous work demonstrated that increases in the tetramer/CD79b ratio are due to increased surface antibody affinity for antigen related to somatic mutations (32). Examples of this analysis are shown in (Figure 5). IgM^+^ S1-RBD tetramer–binding naive B cells from a prevaccination sample, which likely expressed unmutated antibodies (33), had an S1-RBD tetramer mean fluorescence intensity (MFI) (Figure 5A) that was 1.1 times greater than the CD79b MFI (Figure 5B), while IgG^+^ S1-RBD tetramer–binding memory B cells from a 28-week postvaccination sample had a tetramer/CD79b ratio of 6.2. On average, we found that the IgM^+^IgD^+^ S1-RBD tetramer–binding naive cells from participants before vaccination had an S1-RBD tetramer/CD79b ratio of 2.1 (Figure 5, C and D), which increased for the memory B cells in the control group to 4.7 and 6.8 by 8 and 16 weeks after vaccination, respectively, and then increased slightly to 7.1 at 28 weeks. The S1-RBD tetramer/CD79b ratio of the memory B cells in the IBD group also increased but to a lesser extent than controls, reaching values of 4.0, 4.6, and 4.9 by 8, 16, and 28 weeks after vaccination. At 16 weeks, the ratio for the control group was significantly greater than that of the total IBD group or the subsets of TNF-blocked or other immunosuppressive drug–treated patients within the IBD group. A third vaccination, however, increased the average ratio for memory B cells in the IBD group to the 7.0 value observed for the memory B cells in controls. These data are consistent with the memory B cells in both groups undergoing progressive affinity maturation after the second vaccination, but with the memory B cells in the IBD group improving affinity less than those in the control group. However, the defect in memory B cell affinity maturation for most participants in the IBD group was corrected by a third vaccination.

## Discussion

The goal of this study was to identify possible postvaccination defects in antigen-specific B cells in patients with IBD. We found that patients with IBD on immunosuppressive therapies, primarily TNF blockers, produce lower amounts of S1-RBD antibodies, neutralizing antibodies, and antibody-secreting plasmablasts after mRNA vaccination than healthy controls. This result is in line with the reduction in antibodies observed in TNF blocker–treated patients with arthritis after influenza vaccination (34, 35) and other studies of patients with IBD after SARS-CoV-2 mRNA vaccination (5–7). TNF is required for proper formation of the B cell–rich follicles of secondary lymphoid organs where primary immune responses occur (24, 36, 37). Thus, it is possible that TNF inhibitor–treated patients have disorganized lymphoid organs that do not optimally support antigen-driven activation of B cells.

TNF is also critical for formation of the follicular dendritic cell network that captures antigens and drives the germinal center reaction that produces affinity-matured memory B cells (24, 36, 37). Although patients with IBD generated normal numbers of memory B cells after vaccination, TNF deprivation–induced impairments in the follicular dendritic cell network could have accounted for the affinity maturation defect that we observed in this population. It is important to note, however, that reductions in antibody production, plasmablasts, and memory B cell affinity maturation were also observed in patients with IBD treated with agents that block lymphocyte migration to mucosal tissues or inhibit IL-12 and IL-23 or cell division but do not interfere directly with TNF. It is therefore possible that secondary effects of IBD, for example, alteration of the gut microbiome (38), which has been reported to suppress vaccine responses (39), could have accounted for the defects that we observed. This possibility is unlikely, however, given that the disease was well controlled in the patients in our study. It is more likely that the defect in memory B cell affinity maturation in patients with IBD treated with immunosuppressants that target molecules other than TNF reflects the fact that the germinal center reaction depends on many factors.

Our finding that a third vaccination enhances the generation of affinity-matured memory B cells, which could generate plasmablasts that secrete high-affinity antibodies after infection, suggests that a third shot could provide patients with IBD with better immunity to SARS-CoV-2. This happens despite the ongoing use of advanced IBD therapies that should still be affecting the germinal center and demonstrates that repeated doses of mRNA vaccines can overcome the lack of TNF-supporting stromal cells in germinal centers. However, one caveat to our study is that we do not include data on the response of healthy individuals to the third vaccination, because the third vaccination was not yet offered to healthy individuals at time of data collection. Thus, it is possible that we could have seen even greater improvement in affinity maturation in the memory B cell population in healthy controls with repeated vaccination.

As neutralizing antibodies wane after vaccination, the persistence of memory B cells can provide ongoing protection from severe illness. Despite decreases in plasmablast formation, serum antibody titers, and memory B cell affinity maturation, the frequency and stability of memory B cells in patients with IBD was similar to that of healthy controls. Although our study was too small to discern whether patients with IBD are more susceptible to breakthrough infections than healthy controls, it provides a reasonable basis for the policy that these individuals receive a third dose of mRNA vaccine.

## Methods

### Human participants.

Thirty healthy volunteers and 42 patients with IBD on immunosuppressive therapies, all SARS-CoV-2-naive, were followed at the time of and up to 6 months after vaccination with BNT162b2 (Pfizer-BioNTech) (40) or mRNA-1273 (Moderna) (41) mRNA vaccines. Much of the data from healthy volunteers was from another study by our group (10). Patients with IBD were recruited from the University of Minnesota IBD Program or referred from several community gastroenterology practices in the Minneapolis—St. Paul metropolitan area. Medical history was obtained from clinical charts and patients during their research visits. All scheduled blood draws for this study were performed in the research clinic.

### S1-RBD tetramer and decoy production.

An S1-RBD–6xHis tagged protein was generated, purified, and biotinylated as described in Pape et al. (10). Tetramer was prepared by mixing S1-RBD-biotin with of streptavidin-AF647 (SA-AF647) at a ratio of 4:1. The SA-AF647 was added in 4 portions 20 minutes apart and then the final concentration of SA was adjusted to 1 μM. The SA-PE-AF647 decoy was prepared by conjugating AF647 to SA-PE using a kit from Invitrogen (Alexa Fluor 647 Protein labeling kit, A20173), adjusting the SA concentration to 1 μM, and incubating with a molar excess of free biotin to block any free sites on the SA moiety.

### Human PBMC preparation.

Peripheral blood was collected into several 8 mL CPT Mononuclear Cell Preparation Tubes Sodium Citrate (BD, 362761) and stored at 4°C for up to 24 hours until processing. Tubes were then centrifuged at 1700*g* for 20 minutes at 25°C, and 1 mL aliquot of plasma was collected. The remaining PBMCs were removed by aspiration, washed twice with MACS buffer (PBS containing 0.5% fetal bovine serum and 2 mM EDTA), and suspended in 50 μL MACS buffer containing human Fc block (BD, 564219) prior to staining.

### Cell enrichment.

PBMCs were incubated with 10 nM decoy SA-PE-AF647 for 10 minutes and then with 5 nM S1-RBD/SA-AF647 tetramer for 45 minutes at room temperature. The samples were washed and incubated with 25 μL Cy5/AF647 antibody-conjugated MicroBeads (Miltenyi, 130-091-395) for 15 minutes at 4°C, washed again, and then passed over magnetized Miltenyi LS columns. The cells that flowed through the columns were saved. The columns were then washed 3 times and removed from the magnetic field to allow elution of the bound cells in 5 mL sorter buffer. Fluorescent beads (AccuCheck, Life Technologies, PCB100) were added for the purpose of calculating the number of live lymphocytes.

### Flow cytometry.

Column bound or flow-through samples were stained with a Ghost Red 710 viability dye (TonBo, 13-0871-T100) and fluorophore-conjugated antibodies specific for CD3 (OKT3, Invitrogen, 47-0037-42)), CD14 (61D3, Invitrogen, 47-0149-42), CD16 (CB16, Invitrogen, 47-0168-42), CD19 (HIB19, Biolegend, 302242), CD20 (2H7, BD, 563782), CD21 (B-ly4, BD, 562966), CD27 (O323, Biolegend, 302834), CD38 (HB-7, Biolegend, 356620), CD11c (B-ly6, BD, 526393), CD79b (CB3-1, Biolegend, 341404), IgD (IA6-2, Biolegend, 348210), IgM (MHM-88, Biolegend, 314524), IgA (Southern Biotech, 2050-02), and IgG (G18-145, BD, 564230); washed in sorter buffer; and fixed in 250 μL BD Cytofix/Cytoperm. Flow cytometry was performed on a BD Fortessa X20 and analyzed with FlowJo software (Tree Star).

### Calculation of total numbers of S1-RBD tetramer binding cells per million B cells.

The total number of S1-RBD tetramer–binding B cells per million B cells was calculated by dividing the total number of S1-RBD–specific cells in the sample by the total number of single, live, CD19^+^ B cells in the entire sample (bound and flow through) and multiplying by 1 million. The frequency of CD20^–^ plasmablasts and CD20^+^ memory B cells was then multiplied by the total S1-RBD tetramer–binding cells per million B cells to obtain the total plasmablasts and memory B cells per million B cells. Because plasmablasts tend to have less surface BCR than memory B cells, some S1-RBD tetramer–labeled plasmablasts did not bind to the columns and passed into the flow-through fraction. These plasmablasts still had detectable surface BCR and could be detected by flow cytometry in the flow-through fraction. Therefore, at times when plasmablasts were present (2 weeks after the first vaccination or 1 week after the second or third vaccinations), the total numbers of S1-RBD tetramer–binding plasmablasts in the flow through were added to the total numbers in the bound fraction to obtain the total number of S1-RBD tetramer–binding B cells. In some cases, the frequency of IgG^+^ or IgA^+^ cells was multiplied by the total number of S1-RBD tetramer–binding cells per million B cells to calculate the number of S1-RBD tetramer–binding cells of each isotype per million B cells.

### S1-RBD ELISA.

Plasma samples were serially diluted in 96-well plates coated with 3 μg/mL S1-RBD in PBS and blocked with 1% BSA. Plates were incubated at 37°C for 1 hour with horseradish peroxidase–labeled antibodies specific for human Ig heavy and light chains (Invitrogen/Thermo Fisher Scientific, 31412) and developed with KPL ABTS Peroxidase Substrate (SeraCare, 5120-0043). Each plate contained a positive and negative serum as a reference. An ELISA plate reader was used to measure the optical density (405 nm) of each well. Titers were calculated as the reciprocal of the dilution that gave a half-maximal optical density value.

### SARS-CoV-2 virus neutralization assay.

The SARS-CoV-2 virus neutralization assay was performed as previously described (42). Vero E6 cells were added to 96-well plates. Serially diluted plasma samples were mixed for 1 hour with mNeonGreen SARS-CoV-2 virus at 37°C. The mixture was then added to the Vero E6 cells and incubated at 37°C for 24 to 26 hours before fixation in 4% paraformaldehyde. The fixed cells were washed with PBS, and the virus-derived fluorescence signal was determined on a plate reader. Percent maximal infection was determined as the ratio of the fluorescent signal from a given sample to the maximal signal for untreated controls in the same plate. The plasma dilution that neutralized 50% of mNeonGreen fluorescence was determined by logistic regression and defined as the neutralizing antibody titer. Samples that did not achieve 50% inhibition were assigned a titer of 0.5 of the highest plasma dilution tested.

### Statistics.

Statistical significance between the log_10_ cell per million values or S1-RBD/CD79b MFI ratios for individual samples from different groups was analyzed by 2-tailed Student’s *t* tests or ordinary 1-way ANOVA and Šidák’s multiple comparison tests using Prism version 9. The exact values of *n* are stated in the figure legends. Only samples that contained at least 40 S1-RBD tetramer–binding cells were included for analyses of memory B cell subsets. One value was excluded as described in the legend to (Figure 5). Virus neutralization titers were determined using Prism 9 (GraphPad) software. *P* values of less than 0.05 were considered significant.

### Study approval.

The study protocol was reviewed and approved by an independent Institutional Review Board at Advarra Inc. (Columbia, Maryland, USA), with permission from the University of Minnesota. Written informed consent was obtained from participants prior to participation.

## Author contributions

KAP, MKJ, and AK conceptualized the study. KAP, MKJ, and AK curated the data. KAP and MKJ provided formal analysis. MKJ, AK, and TWS acquired funding. KAP, PDK, and WEM provided investigation. KAP provided methodology. MKJ and AK administered the project. TD, AJK, DK, CE, MM, SL, CTG, BPV, ES, TDB, CE, JR, and SS provided resources. MKJ, AK, and RAL provided supervision. PDK, CE, and SS provided validation. MKJ provided visualization. MKJ and KAP wrote the original draft. All authors reviewed and edited the manuscript.

## Figures and Tables

**Table 1:**
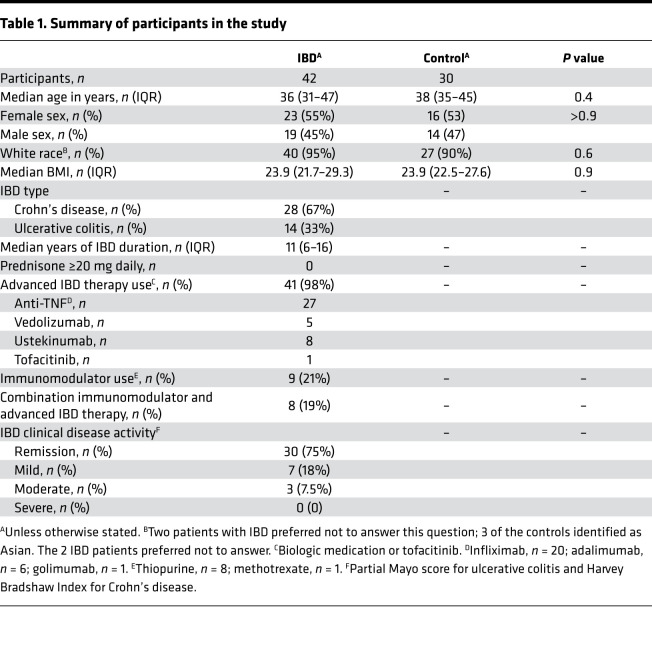
Summary of participants in the study

## Version 1 06/22/2022

Electronic publication
